# Improving an intrinsic-plus movement pattern with a reversed knitted hand function enhancement glove: a case report

**DOI:** 10.3389/fresc.2026.1838253

**Published:** 2026-07-15

**Authors:** Hikaru Mochizuki, Tomoo Mano, Shota Sawamoto, Maya Shinohara

**Affiliations:** 1 Department of Rehablitation Medicine, Teikyo University Chiba Medical Center, Ichihara, Japan; 2 Department of Neurology, Nara Medical University, Kashihara, Japan

**Keywords:** hand strength, intrinsic plus, knitted hand function enhancement glove, motor learning, orthotic devices

## Abstract

This report describes a case in which orthotic therapy and the reversed application of a knitted hand function enhancement glove (Nigirukun®) were used to treat an intrinsic-plus-like movement pattern persisting after recovery from wrist drop secondary to radial nerve palsy. The patient was a man in his 20s who developed radial nerve palsy after a traffic accident. Although wrist drop and sensory disturbance improved during recovery, an intrinsic-plus-like movement pattern characterized by metacarpophalangeal joint flexion with proximal and distal interphalangeal joint extension persisted in the left hand, resulting in difficulty in grasping objects, particularly with the ring and little fingers. The intrinsic tightness test showed no evidence of structural shortening, suggesting that abnormal movement was mainly related to a fixed motor control strategy associated with intrinsic muscle overactivity. Initial treatment consisted of thermotherapy, range-of-motion exercises, and orthotic therapy designed to maintain the interphalangeal joints in flexion. To modify the flexion onset pattern during grasping, a knitted hand function enhancement glove was applied in a reversed orientation to assist interphalangeal joint flexion during the early phase of grasping, thereby reducing intrinsic muscle-dominant activation. Immediately after the introduction of the reversed glove, the patient reported improved ease of grasping, accompanied by increases in grip strength and active motion indices. By hospital day 59, total passive motion showed little change; however, improvements in finger flexor strength, active movement measures, and patient-reported outcomes were observed, suggesting that functional recovery may have been related more to changes in motor strategy than to structural joint changes. These findings suggest that the reversed use of a knitted hand function enhancement glove may support sensorimotor re-education and facilitate the relearning of coordinated flexion patterns in daily activities.

## 1 Introduction

Grasping movements of the hand depend heavily on the coordinated balance between extrinsic and intrinsic muscles. Shortening or contracture of the intrinsic muscles as well as disruption of the strength balance between the intrinsic and extrinsic muscles may result in a typical intrinsic-plus deformity (metacarpophalangeal [MCP] joint flexion with proximal and distal interphalangeal [PIP/DIP] joint extension) or its opposite deformity. Trauma, prolonged immobilization, and disuse or adhesions following nerve palsy are known causes of intrinsic muscle contracture, which may markedly impair grasping, pinching, and fine motor functions (1).

Recent studies suggest that compensatory hand use following peripheral nerve injury may alter movement patterns (2). Furthermore, neurophysiological research has shown that altered movement patterns after peripheral nerve injury may lead to reduced sensory feedback and decreased use of efferent motor signals, influencing central regions, such as the primary somatosensory cortex and supplementary motor area, and contributing to cortical reorganization (3–5). Novak et al. (6, 7). emphasized that recovery after nerve injury requires sensorimotor re-education, in which the brain relearns and reorganizes appropriate sensory input and motor output patterns, and that range-of-motion (ROM) exercises alone may be insufficient for functional recovery.

Based on these findings, an intrinsic muscle-dominant movement pattern following peripheral nerve injury may represent not only structural intrinsic muscle shortening or contracture, but also a compensatory motor control strategy.

Surgical intervention may be indicated in cases of severe intrinsic muscle contractures. However, in mild cases, conservative management—including ROM exercises, splinting to maintain functional positioning, and sensorimotor re-education based on normal movement patterns—may be required (1).

Nigirukun (Nigirukun®, Mikasa Co., Ltd., Japan) is a knitted hand function enhancement glove originally developed for low-impact rehabilitation and elastic-assisted movement, which has been used for patients with sarcopenia or prolonged hospitalization (8, 9).

We present a patient with finger extension contracture that persisted following a wrist drop secondary to radial nerve palsy. In addition to orthotic therapy, a knitted hand function enhancement glove was applied in a reversed orientation, showing potential for immediate improvement.

We hypothesized that the reversed glove application assists finger flexion during grasping, thereby facilitating relearning of a more coordinated grasping pattern.

This case highlights the potential role of reversed glove application as a practical method for sensorimotor re-education in patients with persistent intrinsic-plus-like movement patterns.

## 2 Methods

### 2.1 Case description

Following a motor vehicle accident, a man in his 20s sustained a cerebral contusion, multiple fractures of the right pelvis and lower extremities, and a left humeral shaft fracture complicated by radial nerve palsy with wrist drop. Because impaired consciousness persisted for approximately 3 months after the injury, active ROM exercises were difficult to perform. A cock-up splint was prescribed; however, wearing time was limited because of skin abrasions caused by sensory disturbance.

During daily activities, compensatory pinching using the thumb and index finger in the wrist-drop position was frequently observed, and extension-dominant finger use became habitual. After transfer to a rehabilitation hospital and repeated readmission to our hospital for right total hip arthroplasty and treatment of an abscess, radial nerve palsy, sensory disturbance, and wrist extension limitation improved. However, during pinching with the left hand, an intrinsic-plus movement pattern characterized by MCP flexion with PIP/DIP extension persisted.

Occupational therapy included active and passive ROM exercises for the fingers. On day 1, total passive motion (TPM), manual muscle testing, and grip strength were evaluated (Table 1). In the left hand, extension contracture associated with prolonged extension positioning and an intrinsic-plus movement pattern remained, particularly in the ring and little fingers, where ROM limitation and muscle weakness were evident.

**Table 1:** Patient evaluation (day 1–59).

Assessment item	Day 1	Day 30〈Before the introduction of glove〉	Day 30〈Immediate effect after the introduction of gloves〉	Day 37	Day 59 (Gloves not used)	Day 59 〈When using gloves〉
MMT(MCP.PIP.DIP) 4th	5.3.4	5.4.5	N/A	N/A	5.5.5	N/A
5th	5.2.4	5.2.4	N/A	N/A	5.4.5	N/A
Left grip strength (kg)	17	20	21.5	N/A	26	26
TPM(°) 4th	210	280	N/A	N/A	280	N/A
5th	140	260	N/A	N/A	250	N/A
%TAM (%) 4th	N/A	76	82	N/A	92	92
5th	N/A	64	68	N/A	80	80
FTPD (cm) 4th	N/A	0.7	0.5	N/A	0	0
5th	N/A	1.8	1.6	N/A	1	0.9
MAL-AOU	N/A	2.14	N/A	3.78	4	N/A
MAL-QOM	N/A	3	N/A	3.5	4.07	N/A
HAND20 (points)	N/A	20	N/A	N/A	18	N/A

N/A; Not Available.

The patient was independently ambulatory using Lofstrand crutches; however, impaired grasping with the ring and little fingers interfered with tasks such as opening jars and holding a toothbrush. He wished to return to work, driving, and hobbies including darts and golf. Therefore, restoration of functional use of the affected hand, rather than use only as an assistive hand, was established as the treatment goal.

An intrinsic tightness test demonstrated preserved joint mobility, suggesting that the primary cause was fixation of the movement pattern owing to intrinsic muscle overactivity rather than structural shortening. Treatment included ROM exercises after thermotherapy, stretching with IP flexion and MCP extension, and peg-based sensorimotor re-education. Orthotic therapy aimed at reducing intrinsic muscle overactivity and improving PIP/DIP joint ROM was also considered. Written informed consent for publication was obtained from the patient.

### 2.2 Outcome measures

Manual muscle testing (MMT) of finger flexion was performed using the standard 6-point Medical Research Council grading system.

Grip strength was measured using a Smedley-type dynamometer with the shoulder adducted and elbow extended.

FTPD was measured as the distance between the fingertip and distal palmar crease during maximal active flexion.

%TAM was calculated according to the American Society for Surgery of the Hand criteria.

The Japanese version of the Motor Activity Log (MAL) and HAND20 were administered to assess subjective upper-limb use and hand function. MAL was administered using a structured interview, and HAND20 was self-administered by the patient.

### 2.3 Intervention course

#### 2.3.1 Phase 1: fabrication and modification of a finger flexion orthosis

The objectives were to maintain the IP joints in flexion during grasping to reduce intrinsic muscle overactivity, improve the DIP and PIP joint ROM, avoid pain or skin redness after several hours of wear, and allow independent donning of the orthosis.

**Figure 1:**
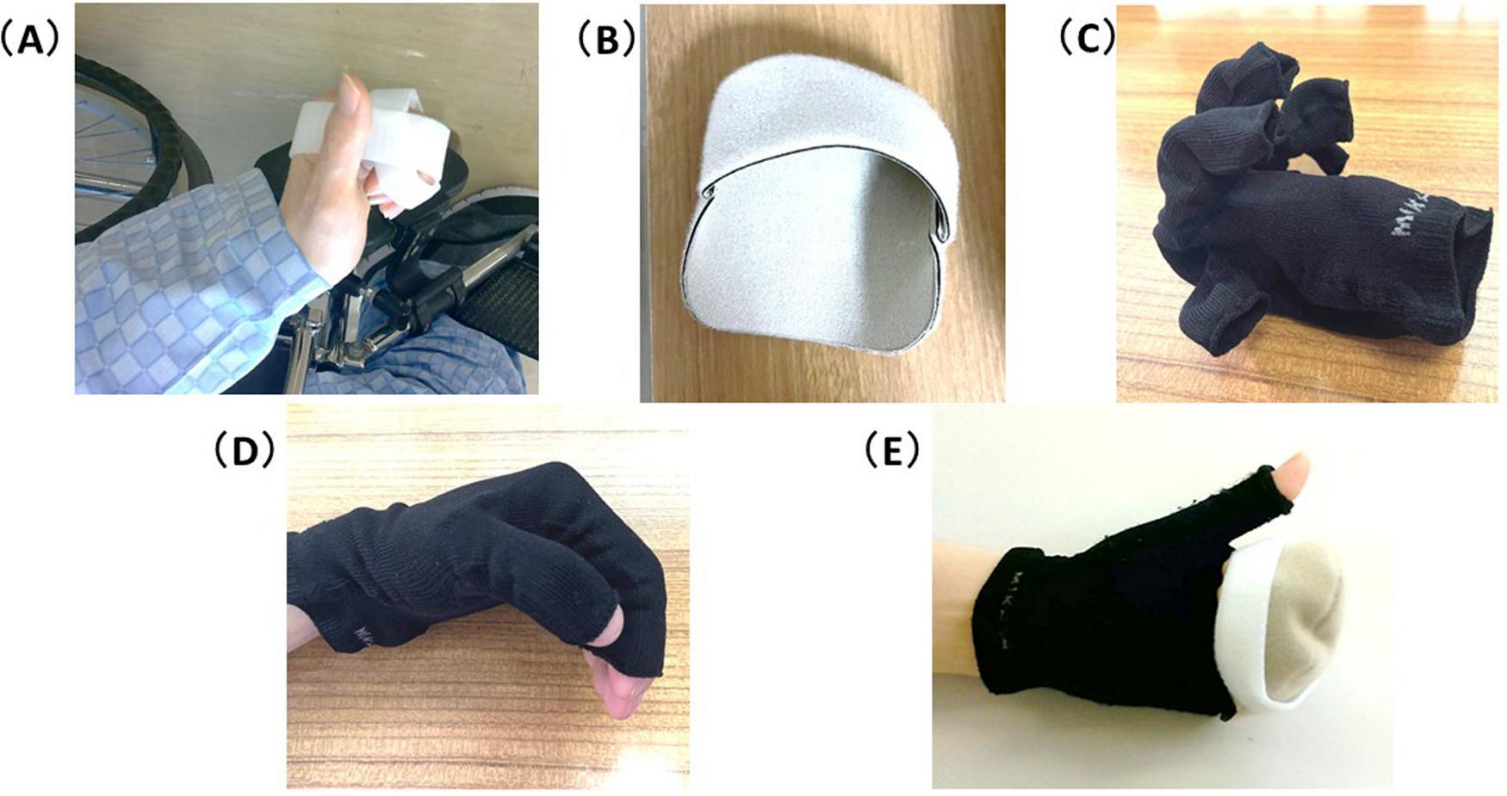
Images of orthotic devices used to improve hand function. **(A)** Soft-splint flexion orthosis. **(B)** Sack-type orthosis made of neoprene. **(C)** Hand function enhancement glove. **(D)** Hand in a relaxed position while wearing the glove. **(E)** Hand wearing the knitted hand function enhancement glove and neoprene orthosis.

An orthosis using soft splint material to fix the PIP and DIP joints in flexion with Velcro was fabricated; however, skin redness and mild pain occurred. Therefore, the design was modified by removing the portion covering the MCP joints and dividing it to cover the middle and proximal phalanges, securing it with Velcro (Figure 1A).

After one week of use, difficulty in maintaining finger flexion occurred because loosening and displacement developed while fastening the Velcro. Therefore, the need for a simpler donning method and materials less likely to irritate the skin was identified.

#### 2.3.2 Phase 2: fabrication of a neoprene finger flexion orthosis

Considering the difficulties identified in Phase 1, a sack-type orthosis made of neoprene, providing superior elasticity and pressure distribution, was fabricated from day 22 (Figure 1B). The structure enveloped the IP joints in flexion. After training, the patient was able to don the orthosis independently.

Although no skin problems occurred with prolonged wear, insufficient rigidity sometimes led to sagging and inadequate maintenance of IP joint flexion, which required additional Velcro fixation. Because the fingers were prepositioned in flexion by the neoprene material, the patient could secure the Velcro independently and achieve fixation at maximal flexion.

The intrinsic-plus movement pattern during object grasping persisted. Therefore, the effect of correcting the flexion onset pattern was limited, and prolonged sensorimotor re-education was considered necessary.

#### 2.3.3 Phase 3: reversed application of the knitted hand function enhancement glove

To correct the flexion onset pattern during grasping and promote repeated sensorimotor re-education during daily activities, the reversed application of a knitted hand function enhancement glove was introduced (Figure 1C).

We hypothesized that reversed wear would allow elastic recoil of the glove to assist MCP and PIP joint flexion, prevent intrinsic muscle overactivity, and suppress the intrinsic muscle-dominant flexion onset pattern (Figure 1D).

Immediately after application (on day 30), the patient reported subjective improvement in grasping and force generation, and the design was evaluated as practical for daily use. Immediate improvements in grip strength, fingertip-to-palm distance (FTPD), and % total active motion were observed (Table 1).

Because of a previous history of abrasions with splint use, glove wear was limited to daytime use under supervision, excluding meals, grooming, and bathing, with a target of ≥9 h/day. A neoprene orthosis was worn as tolerated (Figure 1E). As self-training, stretching combining IP flexion with MCP extension was continuously performed for ≥15 min/day.

After one week, the Motor Activity Log (MAL) Amount of Use and Quality of Movement scores improved beyond the minimal clinically important difference (1.0–1.2) (10), and no significant adverse events were observed. After discharge home, the same training and wearing method were continued. A neoprene orthosis was used only at night for sustained stretching.

On day 59 (Table 1), total passive motion showed no marked changes. However, compared with day 30, manual muscle testing demonstrated improvement, particularly in PIP flexor strength, and active movement measures, including FTPD and % total active motion. Subjective assessments showed improvements exceeding the minimal clinically important difference for MAL, whereas HAND20 scores did not show a marked improvement. While wearing the glove, the patient reported a slipping sensation when grasping objects, most likely because the glove lacked an anti-slip surface, particularly during forceful or bilateral tasks.

**Figure 2:**
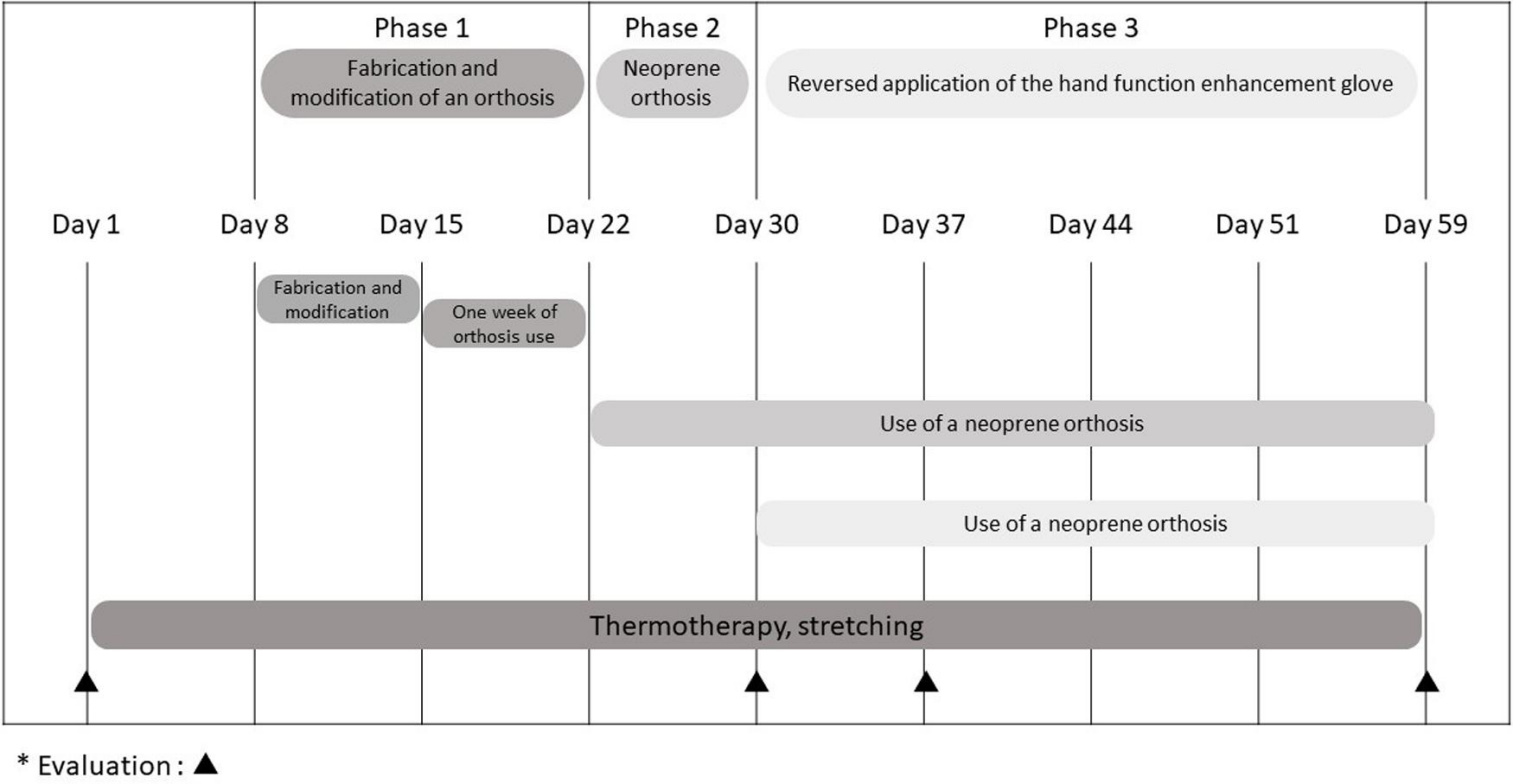
Clinical timeline.

The immediate effects on day 59 showed no change in grip strength or % total active motion with glove wear. The FTPD of the little finger improved to 0.9 cm after application, but no marked overall change was observed. The overall clinical course is presented (Figure 2).

## 3 Discussion

In this case report, the patient's grasping difficulty centered on the ring and little fingers following a wrist drop secondary to radial nerve palsy, and improved after combined orthotic therapy and reversed use of the knitted glove.

Initially, ROM limitations coexisted with a habitual intrinsic muscle-dominant movement pattern during grasping. Orthotic therapy, ROM exercises, and thermotherapy improved total passive motion; however, neither the intrinsic-plus movement pattern nor muscle strength improved markedly. This finding suggests that the condition was not solely caused by decreased flexibility or muscle shortening, but rather by the combined effects of the wrist drop, prolonged immobilization, and compensatory use were likely associated with altered sensory input and maladaptive motor control strategies that persisted even after radial nerve recovery.

The reversed glove application may have assisted IP joint flexion from the early phase of grasping, suppressing intrinsic muscle-dominant flexion onset and promoting the repetition of a more physiological flexion pattern involving the extrinsic muscles, thereby contributing to sensorimotor re-education in a manner that is consistent with previous concepts following peripheral nerve injury.

In normal grasping, extrinsic muscles primarily generate grip force, with IP joints flexing first and intrinsic muscles contributing to MCP flexion in later phases (11–13). When intrinsic muscle overactivity produces simultaneous MCP flexion and IP extension, fingertip wrapping is inhibited and grip strength decreases. In this case, correction of the flexion pattern through glove assistance likely contributed to the improvements in grip strength, FTPD, and % total active motion.

On day 59, passive ROM showed little change, whereas active movement measures improved markedly. Previous reports on intrinsic muscle contracture have primarily focused on structural shortening of the intrinsic muscles, with stretching, splinting, and surgical intervention recommended according to severity (1). However, in the present case, intrinsic muscle extensibility was preserved on the intrinsic tightness test despite the persistence of an intrinsic-plus-like movement pattern during grasping. These findings suggest that even in the absence of severe structural contracture, abnormal motor control and learned compensatory movement patterns may contribute to hand dysfunction, requiring modification of motor strategies and reorganization of movement control.

Furthermore, previous studies have emphasized the importance of sensorimotor re-education and task-specific practice for restoring functional movement following peripheral nerve injury (2, 3, 6). The present case extends these concepts by suggesting that a simple glove may facilitate repetition of a more physiological grasping pattern during daily activities, thereby providing opportunities for continuous motor relearning outside supervised therapy sessions.

Hand function depends on the timing and coordination of multiple muscles during the grasping process (14). Extrinsic muscles possess greater muscle volume and contractile force, contributing to grip and pinch strength, whereas intrinsic muscles contribute to coordination and independent finger control (11, 15). Thus, an improvement in grip strength likely reflects improved extrinsic muscle use.

The smaller immediate effect on day 59 compared to that on day 30 may indicate reduced dependence on elastic assistance and a ceiling effect associated with the formation of a more autonomous motor pattern.

Although the glove was originally intended to strengthen finger extensors, a reversed application may have helped assist with flexion movements through sensorimotor re-education. However, as the glove is made of knitted material, a slipping sensation during object manipulation may occur. Therefore, modifications, such as adding anti-slip material to the palmar surface, may be necessary.

In this case, nighttime sustained stretching using a neoprene orthosis was combined with daytime motor learning. Previous studies have suggested that increasing total end-range time may contribute to improvements in joint range of motion through prolonged low-load stretching (16). Therefore, the synergistic effects of ROM maintenance and learning movement may have contributed to this improvement. Future studies separating orthotic effects from motor learning effects and incorporating quantitative assessments, such as electromyography, to evaluate changes in coordination between intrinsic and extrinsic muscles, are warranted.

This case has some limitations. First, it is a single case report involving natural recovery and concurrent interventions (thermotherapy, ROM exercises, and nighttime sustained stretch using a neoprene orthosis), making it difficult to definitively evaluate the specific effect of the glove alone. Second, there is a need for objective evaluation methods to clearly assess each contributing factor; verification using quantitative functional assessments is required, including study designs that separate the independent effects of orthosis from motor learning effects, and evaluation of changes in coordination between the intrinsic and extrinsic muscles through electromyographic examination. Third, regarding skin-related complications, in cases with severe sensory disturbance or established contracture, skin problems or overload may occur, and careful management is necessary. Additionally, as the glove does not have an anti-slip function, concerns regarding safety remain during forceful tasks. These mechanisms remain hypothetical because no electromyographic or kinematic analyses were performed in this study. Furthermore, whether the modified movement pattern is maintained over the long-term after discontinuing glove use remains unclear. Therefore, future studies involving longer observation periods and a larger number of cases are warranted. To understand the mechanism, objective functional evaluations, such as EMG and kinematic analysis, should be included in the evaluation items.

## 4 Conclusion

Intrinsic-plus movement patterns persist after recovery from wrist drop secondary to radial nerve palsy, and prolonged immobilization may not sufficiently improve by ROM exercises alone.

In this case, the combination of sustained stretching using a neoprene orthosis and flexion assistance with sensorimotor re-education through the reversed use of a knitted hand function enhancement glove improved grasp function.

Therefore, reversed application of the glove may serve as a practical adjunct to promote the relearning of physiological flexion patterns during daily activities.

## Data Availability

The raw data supporting the conclusions of this article will be made available by the authors, without undue reservation.
